# Dominance of *Fagus sylvatica* in the Growing Stock and Its Relationship to Climate—An Analysis Using Modeled Stand-Level Climate Data

**DOI:** 10.3390/plants11192541

**Published:** 2022-09-28

**Authors:** Nina Škrk, Roberto Serrano-Notivoli, Martín de Luis, Katarina Čufar

**Affiliations:** 1 Department of Wood Science and Technology, Biotechnical Faculty, University of Ljubljana, Jamnikarjeva 101, 1000 Ljubljana, Slovenia; 2 Department of Geography, Universidad Autónoma de Madrid, 28049 Madrid, Spain; 3 Department of Geography and Regional Planning, Environmental Sciences Institute (IUCA), University of Zaragoza, 50009 Zaragoza, Spain

**Keywords:** climate, inventory data, *Fagus sylvatica*, climatic suitability, climate modeling

## Abstract

In the future, climate change is expected to affect the spatial distribution of most tree species in Europe. The European beech (*Fagus sylvatica*), a drought-sensitive tree species, is currently distributed throughout Europe, where it is an ecologically and economically important species. In Slovenia, the European beech represents 33% of the growing stock, but such a proportion greatly varies across Europe. Whether such a variation is related to the climate environmental gradients or because of historical or management decisions is an as-yet unexplored question. For this study, we employed the Slovenian Forests Service inventory, where the proportion of beech in the forest stock has been monitored in 341,341 forest stands across the country. Modeled climate data from the SLOCLIM database, calculated for each of the stands, was also used to test the hypothesis that although beech forests have always been influenced by human activity, the dominance of beech trees in forest stands is at least partially dictated by the climate. The results showed the distribution of the main climate variables (annual precipitation, the share of summer and spring precipitation, and annual maximum and minimum temperatures) and how they affect the current dominance of beech trees at the stand level. Due to the large number and variability of forest stands studied, the results should be transferable to better understand and manage the climatic suitability and risks of *Fagus sylvatica*. The modeled data is publicly available in the web repository Zenodo.

## 1. Introduction

**Figure 1:**
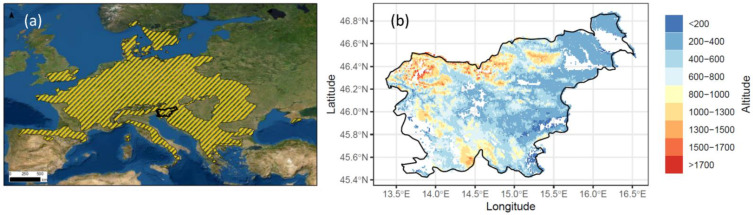
(**a**) Natural range of *Fagus sylvatica* in Europe, with the location of Slovenia marked in black [8,9]; (**b**) natural range of *Fagus sylvatica* across altitudinal gradient (m a.s.l.) in Slovenia [10]. White areas represent forest stands without *Fagus sylvatica*.

The European beech (*Fagus sylvatica*) is one of the most widespread forest tree species in Europe (Figure 1a) and often serves as a model species for studying the response of forests to climate change [1,2,3]. Some recent local studies have shown that the proportion of beech trees is increasing, especially due to the decline of Norway spruce (*Picea abies*) [4]. At the same time, there are numerous reports of local declines in beech numbers [3,5,6,7]. A recent large-scale study of *Fagus sylvatica*, which included a large number of sites covering its geographic and climatic range, showed a current and projected future decline in growth throughout its latitudinal range, especially at its southern limit and in locations where an increase in drought conditions is expected [1]. This suggests that climate influences beech distribution, which depends on the climatic suitability of forest sites.

Sufficient precipitation and moderate temperatures in late spring and summer, especially in June, are particularly important for the growth of beech trees at lower elevations and latitudes, while at higher ones, higher temperatures have a positive effect on their growth [11,12,13,14]. As beech is sensitive to drought and all kinds of weather extremes, climate change also presents a risk for beech trees on sites that currently seem to be optimal for their growth [15]. In Central European conditions, beech reportedly requires at least 600 mm of annual precipitation, with 350 mm in the growing season [14,16]. Summer drought stress reduces beech growth via a reduction in stomatal conductance, sap flow rate, a decrease in the minimum and maximum leaf water potential, and increased fine root mortality [7,17], resulting in decreased vitality and competitiveness [18]. In drier and warmer conditions, beech is also more susceptible to disease [19]. Due to its limited adaptive capacity, beech trees are most vulnerable in marginal sites with climatic conditions that barely allow this species to survive [5,20], although some studies report a higher resistance to drought in the dry range-edge beech populations [15]. Some projections predict a general decline of beech forests by up to 25% in Europe by the year 2100 [1]. Such a scenario would not only cause ecological problems but also considerable economic losses. At the same time, the growth of beech trees could be increased at higher elevations and higher latitudes, where the low temperatures currently limit its growth [2,3,6,11,21]. At lower elevations, beech growth is also expected to be limited due to higher competitive pressure from other species, including oaks [2].

*Fagus sylvatica* is an important forest tree species in Slovenia and grows on a wide variety of sites [22,23] (Figure 1b). However, the actual and potential occurrence of beech trees differ greatly. In numerous locations, beech would prevail if past management practices and exploitation had not converted the areas to agricultural land or coniferous forests [23,24]. The most favorable conditions for beech growth in Slovenia are in a climate zone with a mean annual temperature from 5 °C to 10° C and with a carbonate bedrock [23]. In recent years, beech forest has been expanding its distribution area at an annual rate of 0.24%, especially at lower altitudes [25], often due to the retreat of *Picea abies*. Currently, beech represents 33% of the growing stock of Slovenia [26]. It mostly grows in beech (44%), beech–fir (15%), and beech–oak (11%) forests. Beech trees demonstrate a great ability to grow in different forest associations, sites, climatic regimes, and altitudes from 200 to 1700 a.s.l. [16,27], suggesting that the current conditions are generally favorable for its development. However, due to the numerous human interventions that have affected the natural composition of tree species in forests, it is difficult to determine which areas are most suitable for beech growth.

Defining suitability is difficult since this may reference different biological phases. Dendrochronology has been used to define suitability areas in relation to better growth rates [1], but often, interactions with other species are neglected in this type of analysis. Dendrochronological information is also limited and time-consuming to obtain; as a consequence, detailed information is lacking on the stand scale, the level at which forest management options are applied. Datasets available from forest inventories, by contrast, offer an extraordinary level of detail but have rarely been used for this purpose (defining suitability areas). The Slovenian Forest Service maintains a database on beech tree presence and the species’ contribution to the total growing forest stock for more than 300,000 forest stands [10]. Although climate variability is known to have a major influence on the distribution and growth of beech trees in Slovenia, little is known about how climate may affect the contribution of beech trees to the total growing stock of the forest at stand level.

This study used climate data and information on the percentage of beech trees in the growing stock (%fasy) at a high spatial resolution to test the hypothesis that although beech forests have continuously been influenced by human activity [25], the dominance of beech trees in forest stands is at least partially influenced by climate. To this end, specific objectives were addressed: (1) to create a SLOCLIM_stand dataset, with specific climate information for each individual forest stand in Slovenia, based on the daily climate data available through the modeled database, SLOCLIM, with a spatial resolution of 1 × 1 km [28]; (2) to study the variations of %fasy, seasonal and annual temperature, and precipitation gradients across Slovenia; (3) if the hypothesis is confirmed and climate variations significantly explain the variations in %fasy, we will model such relationships to provide predicted values for %fasy at the stand level across Slovenia.

## 2. Results

### 2.1. Forest Stands and Local Climate Data

**Figure 2:**
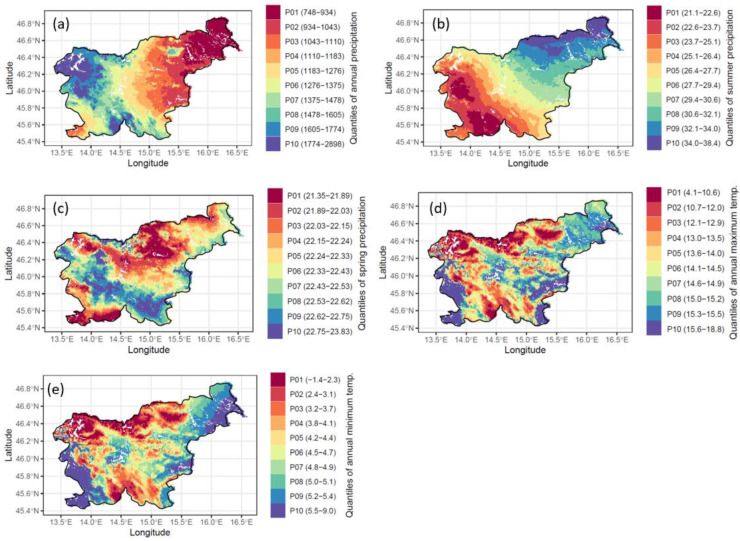
Climate characteristics of the forest stands in Slovenia: (**a**) mean annual precipitation [mm], (**b**) summer precipitation (%), (**c**) spring precipitation (%), (**d**) maximum temperatures (°C), (**e**) minimum temperatures (°C).

The climate dataset, containing the maximum and minimum temperatures and the amount of precipitation on a seasonal and annual level, created for each forest stand, revealed noticeable climatic variability corresponding with the region’s latitudinal, longitudinal, and altitudinal patterns (Figure 2). The highest amount of precipitation is found in the north-western and southern parts of Slovenia (corresponding with the Alpine and Dinaric mountain ridges at the transition between the sub-Mediterranean and temperate climate regimes) reaching or exceeding 1600 mm of annual precipitation (Figure 2a). On the other hand, forest stands with the lowest precipitation prevail in the northeast (in the Pannonian lowland and hills). The share of summer precipitation is the lowest in the western and southern parts of Slovenia (Figure 2b), while the share of spring precipitation is the lowest in the southwest and around Pohorje (Figure 2c). Areas with the highest maximum and minimum temperatures are in the southwest, southeast and the northeast of the country (Figure 2d). The lowest temperatures are in the areas with the highest altitudes (Figure 2e).

### 2.2. Climatic Influences on Beech Population Proportions in Forest Growing Stock

Among the tested generalized mixed models, the full model (selected model) containing all considered and significant variables and interactions was the most accurate model to predict beech dominance across Slovenia, as shown by the Akaike information criterion (AIC) scores (Table 1).

All independent variables and interactions from the model, except the interaction between annual precipitation and annual maximum temperature, were statistically significant in explaining the patterns of beech dominance across Slovenia (Table 2). Mean annual precipitation was the most important climatic factor in explaining beech dominance, followed by spring precipitation. The influence of minimum temperatures was of lesser relative importance, although its effect was significant as well.

**Figure 3:**
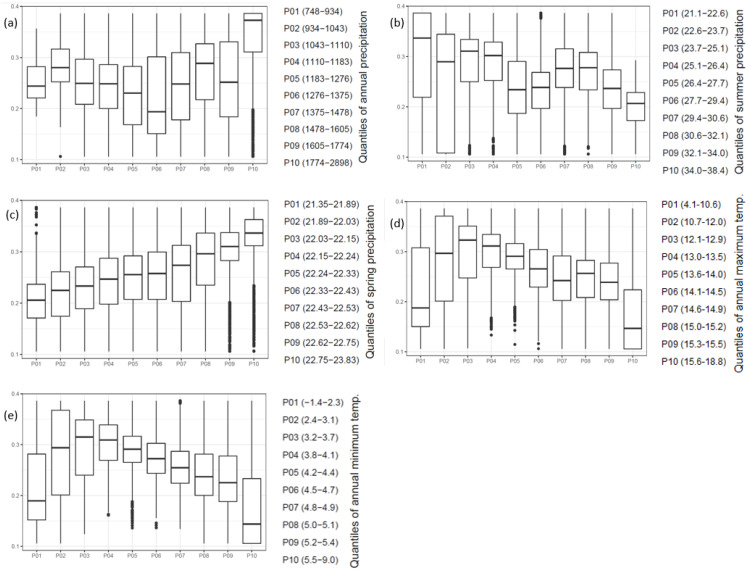
Potential percentage of beech in the growing stock (*y*-axis) in different climatic percentiles (*x*-axis) according to the main variables in the model. Each percentile contains equal number of forest stands with lowest (P01) and highest (P10) values in regard to (**a**) annual precipitation [mm], (**b**) summer precipitation [%], (**c**) spring precipitation [%], (**d**) annual maximum temperature [°C], and (**e**) annual minimum temperature [°C]. Upper and lower limits of boxes represent the 25th and 75th percentiles of data, while the inner horizontal bold line represents the median.

Based on the applied model, boxplots representing the dominance of beech trees across the different climatic gradients were created (Figure 3).

According to the variables used, the highest potential dominance of beech was in the areas with the highest amount of precipitation (more than 1774 mm of annual precipitation) and the lowest in areas with an intermediate mean annual precipitation of 1276 to 1375 mm (Figure 3a). There was a subtle decrease in suitability for beech trees when summer precipitation increased, suggesting that beech dominance decreases when precipitation is concentrated in the summer (Figure 3b).

On the other side, dominance for *Fagus sylvatica* was higher in areas where spring is the main precipitation season (Figure 3c). Although the difference in percentages was small, there was a clear trend of increasing beech dominance predicted with increasing amounts of spring precipitation.

Furthermore, maximized beech dominance is expected in areas where the maximum annual temperature ranges from 12.1 to 12.9 °C (Figure 3d). Then, when temperatures either increase or decrease, the dominance of beech trees declines. Thus, the warmest areas, with a maximum annual temperature from 15.6 to 18.8 °C, showed the lowest dominance for beech trees; these represent the marginal sites for this species.

The effect of the minimum annual temperature showed a similar pattern, and the highest beech dominance was expected in areas where the minimum annual temperature ranges from 3.2 to 3.7 °C (Figure 3e). Marginal sites prevail where the minimum annual temperature does not fall below 5.5 °C.

### 2.3. Distribution of Dominance of Fagus sylvatica over Forest Stands

**Figure 4:**
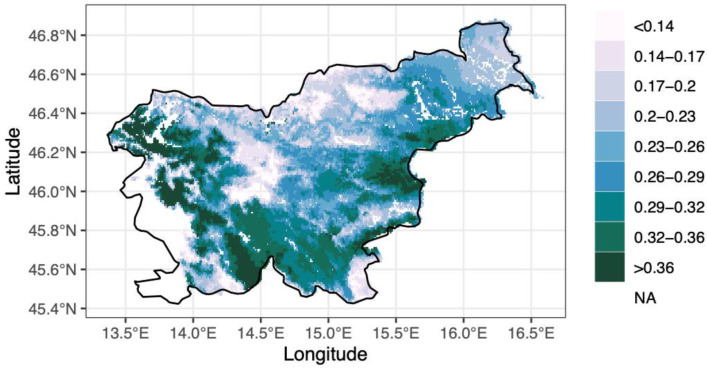
Potential dominance of beech trees in Slovenia. Darker colors represent a higher amount of potential dominance, while lighter colors represent a lower amount.

The applied model helped us to create a map indicating the potential dominance of *Fagus sylvatica* across Slovenia (Figure 4). The potential species dominance also includes those areas where the current beech percentage is low, due to theoretically non-climatic reasons, but with climatic conditions that are suitable for beech survival.

**Figure 5:**
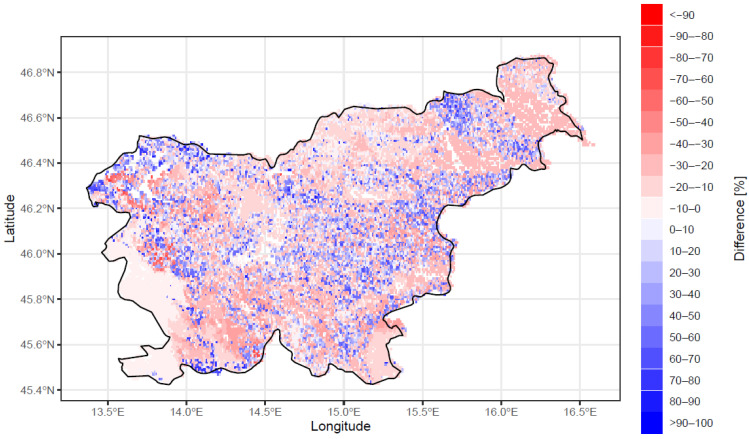
The difference between the observed and potential dominance of beech in Slovenia, as a percentage. Shades of red represent those stands with a lower than potential observed dominance of beech trees, while shades of blue represent the opposite.

In more than 60% of forest stands, the current share of beech trees in the growing stock is lower than its potential share (Figure 5). The difference between potential and actual beech dominance is smallest in the southwest, while, in other parts, the differences are scattered.

## 3. Discussion

Since the growth of *Fagus sylvatica* is predicted to decline in many sites in its natural range, due to climate change [1], we aimed to define those areas that are climatically suitable for beech dominance in Slovenia, taking advantage of the available high-resolution climate and forest inventory data. Using a statistical model, we calculated the potential dominance for particular stands, based on the climatic requirements of beech. For this purpose, we calculated a new dataset of climatic parameters (maximum and minimum temperature and precipitation) for 341,341 forest stands in Slovenia. These stand-level climate datasets can now be used for forest management and research.

Although beech is currently the predominant tree species in Slovenian forests, we confirmed an uneven distribution across the stands with a very low proportion or without beech, mainly in the sub-Mediterranean area (Primorje and Karst), in agricultural areas (Carniola, Ptuj plain, Pomurje (the Mura river basin)), in densely populated and industrialized areas (cities), and at higher altitudes (Julian Alps) (descriptions of the geographical units follow those offered by Perko et al. [29] and Perko and Ciglič [30,31]). However, there are many forest stands with suitable climatic conditions for beech where it does not currently grow, or where its proportion is below the potential presence. Possible reasons for the low proportion, besides climatic conditions, are also other environmental (e.g., unfavorable soil conditions—compacted or dry shallow soil) and anthropogenic (current and past human impacts on forests) conditions. In the past, beech forests were over-exploited by humans [25,32], trees were comprehensively felled for firewood and charcoal production, and the stands were affected by the large-scale removal of forest litter, which had an impact on soil depletion. All this led to the retreat of beech forests. Many potential beech-growing sites were converted to agricultural land; at the same time, *Picea abies*, which was considered the most economically profitable tree species, was favored over beech trees. This all has an effect on the current dominance of beech, although contemporary forest management practices are focused on the nature-based forestry management used since the 1970s, which resulted in a higher proportion of beech in forests [25,33].

According to our results, the distribution of beech dominance in Slovenia, besides historical management practices, is also dependent on climatic conditions. The calculation of potential dominance showed that it is highest in areas with annual maximum temperatures between 12.1 and 12.9 °C, minimum temperatures of 3.2 to 3.7 °C, and annual precipitation between 1700 and 2900 mm, which agrees with the common knowledge [23]. Areas where the share of spring precipitation is small and summer temperatures reach high values are confirmed to be marginal for beech growth; high temperatures, which are associated with low precipitation, negatively affect beech dominance. Higher potential dominance is expected in areas where spring is the main precipitation season, as opposed to the summer share of precipitation. This is related to the physiological processes driving leaf unfolding, cambium reactivation, and the onset of wood and phloem production, which all occur in spring (April–May), with variations depending on the particular site conditions [12,13,20,34]. Therefore, the most climatically favorable conditions for beech dominance are in the northwest (southern parts of the Julian Alps at low elevations, and the Trnovo Forest Plateau), the south (Dinaric Alps), and the eastern part of Slovenia (Kozjansko). The conditions in the southwest (Primorje and Karst) and in the southeastern parts of Slovenia with high temperatures and lower precipitation [28,35] result in a lower beech dominance and represent marginal sites for beech forests.

Based on the model presented in this paper, large areas where climatic conditions would allow a higher share of beech forest than is currently the case can be found in Pomurje, the only flatland macroregion in the country where agricultural landscape prevails [36], the Dinaric Alps, and the Koroška region. Of special interest are the numerous scattered areas where beech can survive in favorable micro-locations, as shown by our study. Although forest cover in the north-east is 29% [37], most of the forest patches and forest fragments in this area are isolated and have a minor depth of interior forest area [36]. The northeast also receives the lowest amount of precipitation in the country [38], but the annual amount is rarely below 600 mm, even in the driest micro-locations. Therefore, water availability per se does not seem to be a limiting factor for beech growth in Slovenia; different amounts within the precipitation range do not seem to have many impacts on beech dominance. More important than the total annual amount is the distribution of precipitation, especially in spring, as shown by this study. The Dinaric Alps have the potential for higher beech dominance compared to the current share, according to the climatic suitability, due to mild temperatures and abundant precipitation; these regions are close to areas where the potential dominance is very low or is close to zero.

The new dataset of beech dominance developed in this work can help forest managers and owners decide where to choose beech for planting in damaged forests, for forest conversion (replacing declining Norway spruce), and for soil fertility improvement. In the context of the future conditions of climate change and the prediction of beech decline in most of Slovenia [1], our data can help to identify micro-locations where beech will first be affected and also the areas where it will have a high potential to grow well. The presented approach can also be applied for studying the suitability of other tree species, to define areas where particular species should be substituted for more resilient ones, or to define favorable patches for the expansion of a particular species [39]. Although beech growth is generally predicted to decline due to climate change, especially at low altitudes and latitudes in Europe and Slovenia [1], it will remain an ecologically and economically important forest tree species. Due to its wide variety of benefits for ecosystems and the variety of possible uses of its wood, beech should not be neglected as a species in future forest management.

## 4. Materials and Methods

### 4.1. Fagus Sylvatica Cover Data

The natural range of the European beech (*Fagus sylvatica* L.) extends throughout Europe; in Slovenia, this species represents 33% of the growing stock. The data on beech cover in Slovenia can be obtained from the Slovenia Forest Service, which maintains a detailed silvicultural monitoring and planning database. This data is systematically recorded and updated annually [10], and contains the forest stand maps for the most important species, including beech, with an average area of forest stand of 3.5 ha [10]. The dataset includes information on tree species composition, growing stock, and developmental phases at the stand level, which is regularly collected and updated.

**Figure 6:**
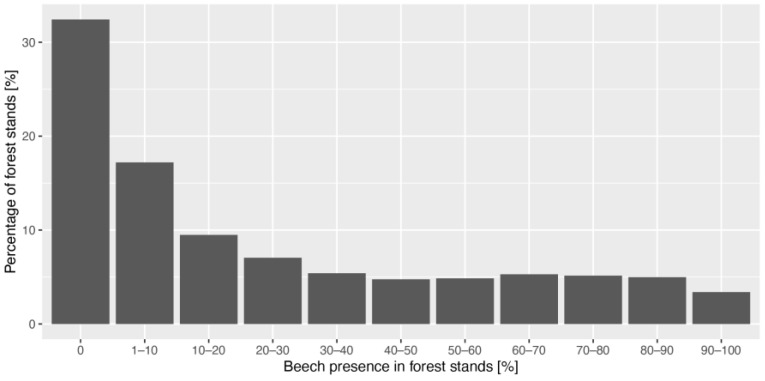
Percentage of *Fagus sylvatica* in the growing stock of 341,341 forest stands in Slovenia.

We used the database with information on forest stands covering all of Slovenia (https://prostor.zgs.gov.si/pregledovalnik/, accessed on 30 March 2021) and extracted data on the percentage of *Fagus sylvatica* volume (m^3^/ha) in the growing stock, compared to other tree species. The data show that *Fagus sylvatica* is generally distributed over most of Slovenia in various and scattered proportions and that areas also exist without beech trees. Some 65.5% of the forest stands contain less than 30% of beech trees in the growing stock, while approximately 35% of the sites contain 30–100% of beech (Figure 6). Stands that are close together and in homogeneous climatic conditions have a reasonably variable presence of beech (scattered distribution), which is potentially due to past forest management activities.

### 4.2. Climatic Data

For the 341,341 forest stands, daily climate data for the period 1950–2018 were extracted from the SLOCLIM climate dataset, at a 1 × 1 km resolution containing 20,998 grid boxes [28]. For stands smaller than 1 km^2^, which are dominant at more than 99% of all stands, we took the climatic data from the intersecting grid box, while for forest stands larger than 1 km^2^, we averaged the climatic data from all the overlapping grid boxes. We calculated the annual precipitation totals and mean maximum and minimum temperatures for each stand. Precipitation varied from 748 mm to nearly 2900 mm, mean maximum temperatures from 4.1 to 18.8 °C, and mean minimum temperatures from −1.4 to 9 °C.

### 4.3. Statistical Procedures

We used SKDV40 (representing the percentage of beech in the growing stock of each forest stand) as the dependent variable describing the dominance of beech trees across stands. Since SKDV40 can, theoretically, vary from 0 to 100, the observed values were re-scaled (0 to 1) for model construction. In order to ensure the balanced weighting of each of the independent variables, all of them were standardized before model construction to have a mean = 0 and a standard deviation = 1.

Climatic variables (annual maximum and minimum temperature and precipitation, as well as their squares, logarithmic transformations, and interactions between variables) were used as independent variables to explore their ability to predict the current dominance of beech across Slovenian forest stands. As specific seasonal droughts can also have an impact, variations in seasonal precipitation distribution across the territory were also included in the model by incorporating two additional independent variables, the percentage contribution of summer and spring precipitation, into the total annual precipitation. This seasonal precipitation was calculated through the ratio of summer (sum of precipitation in June, July, and August) or spring (the sum of precipitation in March, April, and May) and annual precipitation.

Maximum temperatures were included because of the previously established impact of temperature extremes on beech trees in Slovenia [21]. Due to the presence of beech trees at higher altitudes, we also included minimum temperatures in the modeling approach. Annual precipitation was included because it is known that beech is susceptible to general water scarcity and drought [20,40].

Then, generalized linear models (GLMs), using a quasibinomial distribution of the errors, were used. The evaluation of the models and the final selection was based on the AIC criteria. The statistical computing environment R was used through the package, lme4 [41].

## 5. Conclusions

Beech is the predominant tree species in Slovenia, yet still, it does not conquer all potential areas that are climatically suitable for it. Using a high-resolution climatic dataset and a comprehensive silvicultural forest inventory database, we calculated climate data for each forest stand and the potential climatic suitability of forest stands for beech dominance. The dataset is available in the web repository, Zenodo: https://doi.org/10.5281/zenodo.6460250 (accessed on 15 August 2022). The results showed that the potential dominance of beech would be highest in areas with annual maximum temperatures between 12.1 and 12.9 °C, minimum temperatures of 3.2 to 3.7 °C, and annual precipitation between 1700 and 2900 mm. We can thereby characterize the marginal areas for beech growth and the patches where it could remain the dominant species. We think that the approach and results of this study can be applied in other countries that have silvicultural data, to define areas where climatic conditions are most suitable for beech dominance and to develop forest management measures, such as planting schemes in favor of beech trees. In the context of climate change, it is not advisable to promote beech trees in areas where temperatures are currently too high and precipitation too low for this species, or where such conditions are predicted for the future, because beech is then not expected to reach the competitive level of other, more drought-tolerant species, such as oak.

## Figures and Tables

**Table 1:** Accuracy assessment of the best-performing models. Goodness-of-fit measures: AIC (Akaike information criterion), logLik (log-likelihood function), deviance (residual deviance), and df.resid (degrees of freedom—residual). M1 includes annual precipitation, annual maximum temperature, and summer precipitation; M2 adds to M1 the squared and logarithmic transformations of its variables; M3 adds on to M2 the interactions between each one. The selected model adds spring precipitation to M3.

Model	AIC	logLik	Deviance	df.resid
M1	359,170	−179,576.2	169,654	341,332
M2	356,600	−178,287.9	167,402	341,329
M3	352,383	−176,174.6	164,714	341,324
Selected model	351,077	−175,518.3	164,026	341,321

**Table 2:** Parameters of the model showing the coefficients with the estimated value (Estimate), standard error (Std. Error), z value, *p*-value (PR (>|z|)), and significance. Legend: tmax—maximum temperature, tmin minimum temperature, pcp precipitation, ann—annual, 2—squared, l—logarithmic, pcp_sum_prop—summer precipitation; pcp_spr_prop—spring precipitation. * (*p* < 0.05); *** (*p* < 0.001).

Coefficients:	Estimate	Std. Error	z Value	Pr (>|z|)	
(Intercept)	−1.31 × 10^3^	1.03 × 10	−127.392	<2 × 10^−16^	***
pcp_ann	−2.33 × 10^4^	6.04 × 10^2^	−38.563	<2 × 10^−16^	***
tmax_ann	1.07 × 10^7^	7.68 × 10^5^	13.911	<2 × 10^−16^	***
tmin_ann	1.00 × 10^7^	1.70 × 10^6^	5.901	3.62 × 10^−09^	***
pcp_sum_prop	−2.08 × 10^2^	1.17 × 10	−17.852	<2 × 10^−16^	***
pcp_spr_prop	1.37 × 10^2^	5.56	24.573	<2 × 10^−16^	***
pcp_ann2	1.28 × 10^4^	3.13 × 10^2^	40.943	<2 × 10^−16^	***
tmax_ann2	−5.32 × 10^6^	3.84 × 10^5^	−13.847	<2 × 10^−16^	***
tmin_ann2	−5.15 × 10^6^	8.52 × 10^5^	−6.049	1.46 × 10^−09^	***
pcp_annl	1.10 × 10^4^	3.12 × 10^2^	35.126	<2 × 10^−16^	***
tmax_annl	−5.37 × 10^6^	3.84 × 10^5^	−13.976	<2 × 10^−16^	***
tmin_annl	−4.89 × 10^6^	8.50 × 10^5^	−5.752	8.82 × 10^−09^	***
pcp_ann:tmax_ann	1.89 × 10^3^	1.22 × 10^3^	1.544	0.122528	
pcp_sumprop:tmax_ann2	1.44 × 10^2^	1.06 × 10	13.637	<2 × 10^−16^	***
pcp_sprprop:tmax_ann2	6.93 × 10	4.63	14.962	<2 × 10^−16^	***
pcp_ann:tmin_ann	−5.40 × 10^3^	1.29 × 10^3^	−4.190	2.78 × 10^−05^	***
pcp_ann2:tmax_ann2	1.15 × 10^3^	6.10 × 10^2^	1.884	0.059558	
pcp_ann2:tmin_ann2	1.47 × 10^3^	6.39 × 10^2^	2.298	0.021535	*
pcp_annl:tmax_annl	−2.40 × 10^3^	6.42 × 10^2^	−3.742	0.000182	***
pcp_annl:tmin_annl	3.06 × 10^3^	6.75 × 10^2^	4.533	5.81 × 10^−06^	***

## Data Availability

The datasets for this study can be found in the web repository Zenodo: https://doi.org/10.5281/zenodo.6460250 (accessed on 15 August 2022).
